# Toward More Inclusive Networks and Initiatives in Innovation Ecosystems: Protocol for a Systematic Review

**DOI:** 10.2196/34071

**Published:** 2022-05-25

**Authors:** Georgia Ntina, Eirini Mavromanolaki, Andreas D Flouris

**Affiliations:** 1 Discovery Foundation Heraklion, Crete Greece; 2 FAME Laboratory Department of Physical Education and Sport Science University of Thessaly Trikala Greece

**Keywords:** stakeholders, investors, best practice, resources, cluster, accelerator, hub, diverse, diversity, innovative, ecosystem, innovation ecosystem, opening-up strategies

## Abstract

**Background:**

Expanding the cooperation and enlarging the participation of more diverse stakeholders within innovation ecosystems will increase their efficiency and capacity to contribute at local, regional, and national levels.

**Objective:**

This paper presents the protocol for a systematic review that will identify “opening-up” strategies of innovation ecosystems for increasing the participation of more diverse innovation stakeholders, particularly from low-innovation countries, during the ecosystem formation period.

**Methods:**

An algorithmic search in 4 databases (Web of Science, Cochrane Library, Scopus, and Social Science Research Network) will be applied based on the PerSPecTIF (perspective, setting, phenomenon of interest/problem, environment, optional comparison, time/timing, and findings) methodology, the Cochrane guidelines for qualitative evidence synthesis, and the PRISMA (Preferred Reporting Items for Systematic Reviews and Meta-Analyses) guidelines. Selection criteria for eligibility include peer-reviewed articles published after December 31, 1999, and containing original data. No restrictions will be placed on the article language and study region, design, or methodology. Methodological strengths and limitations will be assessed using the Critical Appraisal Skills Programme tool. The thematic synthesis method will be adopted, and the GRADE-CERQual tool will be used to assess confidence.

**Results:**

A preliminary search in Web of Science revealed 2758 records. This work is part of the ANGIE project, which was funded by the European Union’s Horizon 2020 research and innovation program (grant 952152) in January 2021. We anticipate that the results of this systematic review will be published in spring 2022.

**Conclusions:**

We anticipate that the outcomes of this systematic review will outline the best practices used by initiatives and networks, as well as their impacts on creating larger and more inclusive ecosystems.

**Trial Registration:**

OSF Registries osf.io/hc62k 10.17605/OSF.IO/HC62K

**International Registered Report Identifier (IRRID):**

PRR1-10.2196/34071

## Introduction

The “innovation ecosystem” is an umbrella term used to describe the common efforts of different stakeholders to achieve innovation [1,2]. Suppliers provide key parts and technologies that are complemented by products and services provided by a variety of other actors, while customers establish demand and capabilities. In this process of joint value creation, companies gain a competitive advantage by appreciating the overall value of the products and services delivered to customers [1,3-5]. Themes including cooperation between actors, creation and acquisition of value by organizations, and ecosystem leadership have received increasing interest from both practitioners and scholars [6]. Nevertheless, the activities and processes taking place during the ecosystem genesis and expansion phases have received limited attention and many relevant knowledge gaps remain [7,8]. Therefore, there is an urgent need to increase our understanding regarding the formation of innovation ecosystems at their early stage and the formation of roles within these structures of collaboration [2].

Expanding the cooperation and enlarging the participation of more diverse stakeholders within innovation ecosystems will undoubtedly increase their efficiency and capacity to contribute at local, regional, and national levels [9,10]. It will also allow innovation ecosystems to capitalize on opportunities arising from lower costs, improved expertise, as well as new markets and technologies [11-13]. Nevertheless, this topic has received limited attention to date despite having a significant impact not only on policies aiming to promote the economic welfare of sectors, regions, and countries but also on innovation research and practice. Therefore, it is crucial to identify effective methods of “opening-up” for innovation ecosystems to broaden the participation of innovation stakeholders, particularly from low-innovation countries and during the ecosystem formation period. This period of ecosystem evolution is the most fragile, as described in recent research [2,7,8]. Therefore, external provision of the necessary conditions, resources, and activities during this period will have the highest impact. Opening-up strategies include collaborative innovation strategies; network-, market-, and crowd-based innovation strategies [14,15]; and any other activities aiming to increase the ecosystem’s inclusiveness by enlarging the participation of more diverse innovation actors and broadening the participation among different types of stakeholders [16-19].

The aim of this systematic review will be to identify opening-up strategies of innovation ecosystems for increasing the participation of more diverse innovation stakeholders, particularly from low-innovation countries, during the ecosystem formation period. The available evidence on this issue has not been summarized to date to provide a comprehensive understanding of how inclusive innovation ecosystems are formed. The research question expressed via the PerSPecTIF (perspective, setting, phenomenon of interest/problem, environment, optional comparison, time/timing, and findings) statement [20] is presented in Table 1.

**Table 1:** Question formulation based on the PerSPecTIF (perspective, setting, phenomenon of interest/problem, environment, optional comparison, time/timing, and findings) framework [20] for qualitative evidence syntheses.

Elements	Question formulation
Per	From the perspective of innovation stakeholders
S	Particularly from low-innovation countries
P	What are the strategies for “opening-up”
e	Within innovation ecosystems
(c)	N/A^a^
Ti	Up to and including ecosystem formation
F	In relation to increasing the participation of more diverse innovation stakeholders

^a^Not applicable

## Methods

### Overview

The planned systematic review will be conducted according to the current Cochrane guidelines for qualitative evidence synthesis [21] and will be reported in accordance with the PRISMA (Preferred Reporting Items for Systematic Reviews and Meta-Analyses) guidelines [22]. The relevant PRISMA 2020 checklist [22] can be found in Multimedia Appendix 1. The review team includes members trained in systematic review methodology. The protocol has been registered in the Open Science Foundation registry. In case any amendments to this protocol are made during the review process, changes and related reasons will be reported in the final article.

We employed the feasible, interesting, novel, ethical, and relevant (FINER) approach [23] to test the applicability of the research question expressed via the PerSPecTIF statement. The outcome of the FINER approach is presented below:

- Feasible: There is an adequate number of studies for inclusion in the systematic review, since a preliminary algorithmic search in the Web of Science database retrieved 2758 records. Also, the technical expertise of the review team, the dedicated time, and the available funding guarantee its successful completion.
- Interesting: The research questions are interesting, as the systematic review is aiming to provide vital information on opening-up strategies of innovation ecosystems to increase the participation of more diverse innovation stakeholders, particularly from low-innovation countries, during the ecosystem formation period. It is crucial to understand these strategies during the genesis of the ecosystem because it is the most fragile during this period of its evolution. Therefore, external provision of the necessary conditions, resources, and activities during this period will have the highest impact.
- Novel: The systematic review will not only confirm previous findings but will also produce new findings for increasing the participation of more diverse innovation stakeholders to be used for future policies and actions.
- Ethical: There are no ethical concerns regarding the current systematic review process, as it will be entirely based on published evidence accumulation.
- Relevant: The research questions are relevant to current knowledge, practices, and policies pertaining to innovation.

### Inclusion Criteria

Studies investigating opening-up strategies of innovation ecosystems for increasing the participation of more diverse innovation stakeholders will be included. We will consider observational studies that provide original data, independently of the design and methodology adopted.

### Exclusion Criteria

We will exclude narrative reviews, systematic reviews, perspectives, opinion articles, and other publications that do not include original data.

### Years Considered

The selected scientific databases will be searched from January 1, 2000, to present. All searches will be updated prior to submission of the final manuscript, in case the date of the initial search is more than 12 months older than the date of submission [24,25]. New records will be screened and evaluated based on the inclusion and exclusion criteria set above.

### Publication Language

We anticipate a large body of the literature on the topic of this systematic review to be in English. No articles will be excluded based on language, but our search will be conducted using English terms. Articles in languages other than the ones spoken by the coauthors of this study (English, Greek, and German) will be translated into English using Google Translate following previous methodologies [26]. If Google Translate does not generate a good translation or if we are in doubt about the translation generated for a non-English manuscript, the paper will be assessed by a native speaker.

### Search Strategy

We will perform a keyword algorithmic search in scientific databases that focus on research topics relevant to our research question. These databases are as follows: Web of Science [27], Cochrane Library [28], Scopus [29], and Social Science Research Network [30].

The search algorithm will be built according to the PerSPecTIF statement [20] described in Table 1 and will be adjusted to the environment of each database. An example of translating the PerSPecTIF statement to appropriate indexing terms based on existing guidelines [21] is shown in Table S1 (Multimedia Appendix 2). A preliminary search on the Web of Science database using these search terms revealed a total of 1301 records. The algorithm is also provided in Multimedia Appendix 2.

In the algorithmic searching we will use Booleans (ie, OR, AND, NEXT, etc) and possibly truncations as well as wildcard characters (*, ?). Finally, we will also check the reference lists of the included studies for potential eligible publications that did not appear in the initial search approach. To comply with the Multi Engine Command Instrument Rating (MECIR) requirement and Conduct of Systematic Reviews in Toxicology and Environmental Health Research (COSTER) recommendations regarding the updating of the searches within 12 months prior to publication of a systematic search [24,25], we will rerun all searches shortly before the final analyses, and any further relevant studies identified will be retrieved for inclusion.

The above-mentioned pilot algorithm was judged by the review team as applicable and appropriate to be used in the official searching procedure. As such, 2 members of the review team, GN and EM, independently conducted the official searching in the selected scientific databases (from the date of their inception to October 2021). The search algorithm was “translated” from one database to another so that it is compatible with the corresponding website’s search engine. ADF confirmed no disagreement between the team members GN and EM in applying the algorithms in all 3 databases.

### Study Selection

The retrieved studies from the searching procedure will be managed using the Rayyan online platform [31]. Two reviewers (GN and ADF) with previous experience in conducting systematic reviews will independently screen the retrieved publications for eligibility. A third reviewer (EM) will act as a referee and resolve any potential conflicts. Then, all reviewers will share their notes to confirm and finalize the selection of studies. All 3 reviewers will be provided with written instructions on the selection process and will undergo pilot testing of the systematic review selection procedures on a small subset of the records retrieved. The methodology of the selection process is as follows:

1. The first step includes removing duplicated papers that will arise from the searching procedure.
2. The second step includes checking the publications against the eligibility criteria based on titles and abstracts. At this stage, we will exclude records that are not relevant and do not fulfil 1 or more of the inclusion criteria listed above.
3. The third step includes checking the full texts of the remaining publications against the eligibility criteria. This step will result in the final list of the eligible publications.

A PRISMA flow diagram will be created to describe in detail the procedures of searching and selection of publications in this systematic review [22,32]. A full list of the excluded articles will be provided in the final systematic review paper.

### Data Extraction

For each eligible publication, an individual data extraction form will be incorporated. Two review team members will independently extract data from the eligible publications and a referee- investigator will make an ultimate decision in case of a disagreement between review team members. A priori pilot data extraction will be used so that the 2 review team members agree on including missing data that were not initially considered or data that do not need to be extracted. Each team member will extract their data in an Excel spreadsheet (Microsoft Corp). In cases where the presented data are unclear in the articles’ full text, the data extraction review team members will contact the corresponding authors via email to retrieve them.

### Assessment of Methodological Strengths and Limitations

According to the existing guidelines [21], we will assess methodological strengths and limitations as a marker of study rigour using the Critical Appraisals Skills Programme (CASP) tool [33]. CASP is a previously published, commonly used, and validated tool to assess methodological strengths and limitations of qualitative studies [21]. Also, it has shown an interrater agreement of >92% [34]. According to the existing guidelines [21], the aim of this assessment will not be to calculate total quality scores but to provide evidence for a discussion on the studies, their “risk to rigour,” as well as whether their methodological limitations may have affected the systematic review findings.

### Data Synthesis

We will use the thematic synthesis method to produce syntheses that can subsequently be integrated with an intervention review or analysis.

### Assessing Confidence

Based on the existing guidelines [21], we will use the GRADE-CERQual tool to assess confidence in the qualitative synthesized findings. This tool evaluates 4 components (relevance, methodological limitations, adequacy, and coherence) to provide an overall assessment of confidence in the synthesized qualitative findings.

## Results

As indicated above, a preliminary search in the Web of Science database revealed 2758 records. This confirms that the systematic search procedure will generate adequate information to achieve the objective of this review. This work is part of the ANGIE project, which was funded by the European Union’s Horizon 2020 research and innovation program (grant 952152). The ANGIE project was initiated in January 2021. We anticipate that the results of this systematic review will be published in spring 2022.

## Discussion

It is anticipated that the outcomes of this systematic review will outline the best practices used by initiatives and networks, as well as their impacts on creating larger and more inclusive ecosystems with a shared, clearly defined purpose. Additionally, this systematic review will shed light on the steps that innovation ecosystems take to implement a solid opening-up strategy, with the goal of enlarging and broadening the participation and commitment of more stakeholders and encouraging collaborative partnering.

Overall, this systematic review aims to uncover the strategies used by previous and existing ecosystems to become inclusive, fostering the participation of stakeholders at all levels of innovation capacity and taking into consideration gender equality and diversity. Even actors who are moderate and modest innovators can be instrumental, particularly in the early phase of an ecosystem formation [9,10]. Therefore, identifying opening-up strategies of innovation ecosystems for increasing the participation of more diverse innovation stakeholders, particularly from low-innovation countries, during the ecosystem formation period will increase the involvement of different stakeholders and maximize an ecosystem’s innovation potential.

## Appendix

Multimedia Appendix 1 PRISMA (Preferred Reporting Items for Systematic Reviews and Meta-Analyses) checklist.

Multimedia Appendix 2 Describing literature search strategy.

## Abbreviations

CASP: Critical Appraisal Skills Programme
COSTER: Conduct of Systematic Reviews in Toxicology and Environmental Health Research
FINER: feasible, interesting, novel, ethical, and relevant
MECIR: Multi Engine Command Instrument Rating
PerSPecTIF: perspective, setting, phenomenon of interest/problem, environment, optional comparison, time/timing, and findings
PRISMA: Preferred Reporting Items for Systematic Reviews and Meta-Analyses
