# Supplementary material for: Toward More Inclusive Networks and Initiatives in Innovation Ecosystems: Protocol for a Systematic Review
Source: JMIR Res Protoc. 2022 May 25;11(5):e34071. doi: 10.2196/34071 (PMC9178449; doi:10.2196/34071)
Supplement: Multimedia Appendix 2 [file resprot_v11i5e34071_app2.docx]

# ANNEX 2 - Literature search strategy

# Title: Towards more inclusive networks and initiatives in innovation ecosystems: a systematic review protocol

Authors:

Georgia Ntina – Discovery Foundation, Greece

Eirini Mavromanolaki – Discovery Foundation, Greece

Andreas D. Flouris – FAME Laboratory, Department of Physical Education and Sport Science, University of Thessaly, Trikala, Greece & Discovery Foundation, Greece.

Corresponding author:

Andreas D. Flouris

FAME Laboratory, Department of Physical Education and Sport Science

University of Thessaly

Karies, Trikala, 42100, Greece

Tel: +30 2431 047 072.

E-mail: andreasflouris@gmail.com

# Search strategy in the ISI Web of Science database

Based on our perspective, setting, phenomenon of interest, environment, comparison, time, findings (PerSPecTIF) statement [20] (Table 1 of main text), we created a keyword algorithm (1-10-2021) by translating the PerSPecTIF statement to appropriate indexing terms (Table S1) based on Cochrane guidelines [21]. A preliminary search on the Web of Science database revealed a total of 1301 records matching the eligibility criteria for this search. The algorithm is provided in the next section.

| **Table S1 \|** Question formulation and associated keywords based on the PerSPecTIF framework [20] for qualitative evidence syntheses. | | |
| --- | --- | --- |
| **Per** | From the perspective of innovation stakeholders | stakeholder*, investor*, foundation*, public, national, regional, local, private, authorit*, organi?ation*, enterpri?e*, professional association*, small-medium enterpri?e*, small medium enterpri?e*, SME, startup*, start-up*, university*, social innovator*, civil society, non-government*, organi?ation* |
| **S** | Particularly from low-innovation countries | low?innovation, high?innovation, lagging, performance, economy, upper middle?income, middle?income, low?income, moderate, modest, Europe, North America, South America, Latin America, Caribbean, South East Asia, East Asia, Western Asia, Oceania, Central Asia, South Asia, Southern Asia, North Africa, Northern Africa, Sub-Saharan Africa, territory*, region* |
| **P** | What are the strategies for “opening-up” | Training*, soft skill* development, networking, coordination structure*, coordination tool*, best practice*, resource*, knowledge, talent*, market*, expertise*, service*, common knowledge asset*, mutual learning, exchange*, pilot scheme* |
| **E** | Within innovation ecosystems | Cluster*, accelerator*, innovation hub*, incubator*, platform*, technology park* |
| **(C)** |  |  |
| **Ti** | Up to and including ecosystem formation | Origin*, genesis, formation*, start, infancy, development, birth, existence, lifecycle, process* |
| **F** | In relation to increasing the participation of more diverse innovation stakeholders | Collaborative network*, co-creation, interconnection*, widening, inclusive, inclusion, gender-responsive, gender equality, gender balance, diversity, race, ethnic*, geograph*, countr*, region*, equit*, justice, language, “limited English proficiency”, underrepresented, enlarge, broaden |

**Keyword algorithm used in the ISI Web of Science database**

((((((((((((((((((((((((((((((((((((((((TI=(stakeholder*)) OR TI=( investor*)) OR TI=( foundation*)) OR TI=( public)) OR TI=( national)) OR TI=( regional)) OR TI=( local)) OR TI=( private)) OR TI=( authorit*)) OR TI=( organi?ation*)) OR TI=( enterpri?e*)) OR TI=( professional association*)) OR TI=( small-medium enterpri?e*)) OR TI=( SME)) OR TI=( startup*)) OR TI=( start-up*)) OR TI=( university*)) OR TI=( social innovator*)) OR TI=( civil society)) OR TI=( non-government* organi?ation*))) OR AB=(stakeholder*)) OR AB=( investor*)) OR AB=( foundation*)) OR AB=( public)) OR AB=( national)) OR AB=( regional)) OR AB=( local)) OR AB=( private)) OR AB=( authorit*)) OR AB=( organi?ation*)) OR AB=( enterpri?e*)) OR AB=( professional association*)) OR AB=( small-medium enterpri?e*)) OR AB=( SME)) OR AB=( startup*)) OR AB=( start-up*)) OR AB=( university*)) OR AB=( social innovator*)) OR AB=( civil society)) OR AB=( non-government* organi?ation*) AND ((((((((((((((((((((((((((((((((((((((((((((((((((((((TI=(low?innovation,)) OR TI=(high?innovation,)) OR TI=(lagging,)) OR TI=(performance,)) OR TI=(economy,)) OR TI=(upper middle?income,)) OR TI=(middle?income,)) OR TI=(low?income,)) OR TI=(moderate,)) OR TI=(modest,)) OR TI=(Europe,)) OR TI=(North America,)) OR TI=(South America,)) OR TI=(Latin America,)) OR TI=(Caribbean,)) OR TI=(South East Asia,)) OR TI=(East Asia,)) OR TI=(Western Asia,)) OR TI=(Oceania,)) OR TI=(Central Asia,)) OR TI=(South Asia,)) OR TI=(Southern Asia,)) OR TI=(North Africa,)) OR TI=(Northern Africa,)) OR TI=(Sub-Saharan Africa,)) OR TI=(territory*,)) OR TI=(region*))) OR AB=(low?innovation,)) OR AB=(high?innovation,)) OR AB=(lagging,)) OR AB=(performance,)) OR AB=(economy,)) OR AB=(upper middle?income,)) OR AB=(middle?income,)) OR AB=(low?income,)) OR AB=(moderate,)) OR AB=(modest,)) OR AB=(Europe,)) OR AB=(North America,)) OR AB=(South America,)) OR AB=(Latin America,)) OR AB=(Caribbean,)) OR AB=(South East Asia,)) OR AB=(East Asia,)) OR AB=(Western Asia,)) OR AB=(Oceania,)) OR AB=(Central Asia,)) OR AB=(South Asia,)) OR AB=(Southern Asia,)) OR AB=(North Africa,)) OR AB=(Northern Africa,)) OR AB=(Sub-Saharan Africa,)) OR AB=(territory*,)) OR AB=(region*) AND ((((((((((((((((((((((((((((((((TI=(Training*,)) OR TI=(soft skill* development)) OR TI=(networking,)) OR TI=(coordination structure*,)) OR TI=(coordination tool*,)) OR TI=(best practice*,)) OR TI=(resource*,)) OR TI=(knowledge,)) OR TI=(talent*,)) OR TI=(market*,)) OR TI=(expertise*,)) OR TI=(service*,)) OR TI=(common knowledge asset*,)) OR TI=(mutual learning,)) OR TI=(exchange*,)) OR TI=(pilot scheme*))) OR AB=(Training*,)) OR AB=(soft skill* development)) OR AB=(networking,)) OR AB=(coordination structure*,)) OR AB=(coordination tool*,)) OR AB=(best practice*,)) OR AB=(resource*,)) OR AB=(knowledge,)) OR AB=(talent*,)) OR AB=(market*,)) OR AB=(expertise*,)) OR AB=(service*,)) OR AB=(common knowledge asset*,)) OR AB=(mutual learning,)) OR AB=(exchange*,)) OR AB=(pilot scheme*) AND ((((((((((((TI=(Cluster*,)) OR TI=(accelerator*,)) OR TI=(innovation hub*,)) OR TI=(incubator*,)) OR TI=(platform*,)) OR TI=(technology park*))) OR AB=(Cluster*,)) OR AB=(accelerator*,)) OR AB=(innovation hub*,)) OR AB=(incubator*,)) OR AB=(platform*,)) OR AB=(technology park*) AND ((((((((((((((((((((TI=(Origin*,)) OR TI=(genesis,)) OR TI=(formation*,)) OR TI=(start,)) OR TI=(infancy,)) OR TI=(development,)) OR TI=(birth,)) OR TI=(existence,)) OR TI=(lifecycle,)) OR TI=(process*))) OR AB=(Origin*,)) OR AB=(genesis,)) OR AB=(formation*,)) OR AB=(start,)) OR AB=(infancy,)) OR AB=(development,)) OR AB=(birth,)) OR AB=(existence,)) OR AB=(lifecycle,)) OR AB=(process*) AND (((((((((((((((((((((((((((((((((((TI=(Collaborative network*,)) OR TI=(co-creation,)) OR TI=(interconnection*,)) OR TI=(widening,)) OR TI=(inclusive,)) OR TI=(inclusion,)) OR TI=(gender-responsive,)) OR TI=(gender equality,)) OR TI=(gender balance,)) OR TI=(diversity,)) OR TI=(race,)) OR TI=(ethnic*,)) OR TI=(geograph*,)) OR TI=(countr*,)) OR TI=(region*,)) OR TI=(equit*,)) OR TI=(justice,)) OR TI=(language,)) OR TI=(“limited English proficiency”,)) OR TI=(underrepresented,)) OR TI=(enlarge,)) OR TI=(broaden))) OR AB=(Collaborative network*,)) OR AB=(co-creation,)) OR AB=(interconnection*,)) OR AB=(widening,)) OR AB=(inclusive,)) OR AB=(inclusion,)) OR AB=(gender-responsive,)) OR AB=(gender equality,)) OR AB=(gender balance,)) OR AB=(diversity,)) OR AB=(underrepresented,)) OR AB=(enlarge,)) OR AB=(broaden)
